# Molecular Dynamics Study of Defect Evolution in Inconel 617 Alloy Under Successive Cascade Irradiation

**DOI:** 10.3390/ma19040732

**Published:** 2026-02-13

**Authors:** Jiwei Lin, Tianyi Hu, Xu Yu, Hai Huang, Yang Ding, Junqiang Lu

**Affiliations:** 1 Shanghai Nuclear Engineering Research & Design Institute Co., Ltd., Shanghai 200233, China; 2 State Key Laboratory of Materials for Advanced Nuclear Energy, Shanghai 200233, China; 3 Key Laboratory of Material Physics, Ministry of Education, School of Physics, Zhengzhou University, Zhengzhou 450001, China

**Keywords:** Inconel 617 alloy, irradiation damage, defect evolution, molecular dynamics, heat pipe-cooled reactor

## Abstract

Inconel 617 (IN617) is a promising structural material for advanced nuclear systems such as heat pipe-cooled reactors, but its fundamental defect evolution under neutron irradiation remains poorly understood. This study employs classical molecular dynamics simulations to investigate the atomic-scale irradiation damage mechanisms in a representative Ni–Cr–Co ternary model of IN617 under successive displacement cascades. The results reveal a near-linear accumulation of Frenkel pairs with dose, with the count increasing by a factor of approximately 24 from the first to the 75th cascade. A critical finding is the stark asymmetry in defect kinetics: interstitials rapidly coalesce into large clusters (with 88.4% of interstitials found in clusters of ≥ 2 atoms after 75 cascades), while vacancies remain predominantly isolated (constituting 68.8% of all vacancy defects). This disparity directly governs microstructural evolution, as interstitial cluster growth drives dislocation loop nucleation, leading to a linear rise in dislocation density to a saturated value of approximately 4.5 × 10^−4^ Å^−2^. The saturated dislocation structure subsequently undergoes continuous reorganization through reactions between partial dislocations. These insights demonstrate that irradiation hardening in IN617 under simulated conditions is governed primarily by interstitial-type defect clustering, providing a crucial mechanistic basis for assessing its performance in radiation environments.

## 1. Introduction

Building upon Gen-IV nuclear technology, heat pipe-cooled reactors (HPCRs) provide inherently safe designs characterized by compactness, simplicity, and reliable autonomous operation [1,2,3]. These attributes present strong potential for deployment in specialized roles such as space propulsion, subsea power modules, and remote microreactors [3,4,5,6]. The long-term feasibility of such systems, however, is fundamentally reliant upon the durability of their constituent structural materials. Considering the heat pipe-cooled molten salt reactor (HP-MSR)—an advanced adaptation of molten salt reactor technology—raising its operating temperature (potentially to 1373 K) represents a highly efficient route to enhance performance in high-temperature applications like hydrogen production [2,7,8]. The conventional choice for molten salt environments, Hastelloy N alloy, is generally restricted to temperatures below 923 K [1,8,9]. Its mechanical capabilities at elevated temperatures are insufficient for the more demanding HP-MSR operational regime. Conversely, Inconel 617 alloy (IN617) displays superior high-temperature strength and sustained mechanical stability, positioning it as a viable alternative [8,9,10,11]. Supporting this, Gao et al. [10] demonstrated that a corrosion-resistant variant of IN617 satisfies key material requirements in molten salts, a finding further corroborated by Lei et al. [11], who reported exceptional tensile properties from room temperature up to 1073 K, exceeding those of Hastelloy N. A critical consideration for deploying IN617 in HP-MSRs is its performance under neutron irradiation [8,9]. While prior research has extensively examined the alloy’s corrosion resistance and mechanical behavior [8,9,10,11], its response to irradiation damage remains largely unaddressed. Consequently, developing IN617 for this application requires a foundational understanding of its irradiation damage mechanisms.

Generally, the interaction of fast neutrons or energetic fission fragments with structural materials in nuclear reactors transfers kinetic energy to lattice atoms, displacing them from their equilibrium sites, creating primary knock-on atoms (PKAs) of varying energies [12,13]. These energetic PKAs trigger localized collision cascades, generating high concentrations of point defects—specifically vacancies and self-interstitial atoms [12]. The resultant defects can subsequently cluster, via diffusion, into extended microstructural entities like stacking fault tetrahedra, dislocation loops, and voids [13,14]. The accumulation of such features drives macroscopic material degradation, manifesting as hardening, embrittlement, volumetric swelling, and phase transformations [14]. Consequently, the kinetics and evolution of point defects under irradiation are central to predicting material performance. Experimentally capturing the initial damage events poses significant difficulties, as the crucial stages of atomic displacement and cascade development unfold over spatial and temporal scales of nanometers and picoseconds, beyond the direct resolution of most analytical instruments [12,14]. This observational gap has established classical molecular dynamics (MD) simulation as an indispensable tool for probing the fundamental behaviors of point defects, including their nucleation, migration, and aggregation [13,15,16]. For decades, MD has successfully provided atomistically resolved, time-dependent visualization of radiation damage mechanisms, operating at precisely these relevant physical scales across numerous material systems [16,17,18,19].

Therefore, in this work, classical MD simulations were employed to systematically investigate atomic-scale defect evolution in a ternary Ni–Cr–Co system representing IN617 under irradiation. Through the simulation of overlapping displacement cascades, we aim to elucidate the fundamental mechanisms governing radiation damage in this alloy. The paper is organized as follows: first, the computational model and cascade simulation methodology are detailed; subsequently, a quantitative analysis of point defect accumulation and clustering is presented, highlighting the distinct kinetic behaviors of interstitials and vacancies; finally, the evolution of the dislocation network—from its formation to saturation and subsequent reorganization—is examined. Through this structured analysis, the study clarifies the microstructural response of IN617 to irradiation, thereby providing foundational insights relevant to its potential use in advanced nuclear environments.

## 2. Simulation Methodology

**Figure 1:**
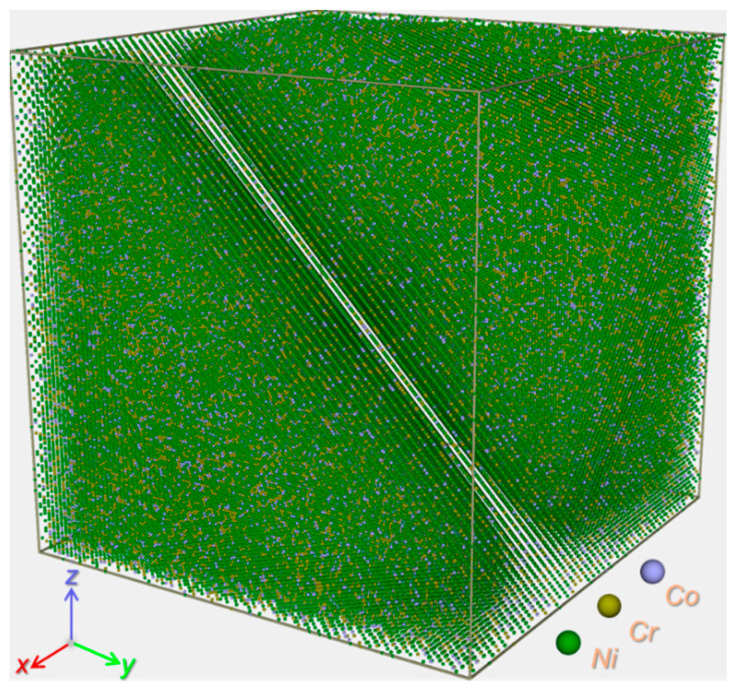
Atomic-scale computational model of the single-crystal ternary Ni–Cr–Co system representing IN617. Atoms are color-coded by element: Ni (green), Cr (brown), and Co (purple).

Classical MD simulations were performed in LAMMPS (LAMMPS, version 29 Aug 2024) [20], with OVITO (version 3.11.3) employed for trajectory analysis and visualization [21]. The computational model comprised a face-centered cubic (fcc) IN617 single crystal (see Figure 1), constructed as a cubic supercell with dimensions of 45a_0_ on each side (a_0_ = 3.52 Å), totaling 364,500 atoms. The simulation cell was oriented with its axes aligned with the principal crystallographic directions [100], [010], and [001]. Cr and Co atoms were randomly distributed on lattice sites at nominal concentrations of 23.62 at.% and 11.14 at.%, respectively, consistent with the bulk composition of IN617 [9,22]. Given that Ni, Cr, and Co constitute the primary alloying elements, the material was represented as a ternary Ni–Cr–Co system—a simplification commonly adopted in comparable simulation studies, which retains the essential physics for a qualitative analysis of radiation-induced damage [7,17,18]. Interatomic forces were described using an embedded atom method (EAM) potential parameterized for Ni–Cr–Co systems [23], supplemented by a Ziegler–Biersack–Littmark (ZBL) screened Coulomb potential to accurately model short-range, high-energy atomic collisions [24]. It should be noted that this model does not include an explicit electronic stopping term. This simplification is justified in the present study, as the selected PKA energy falls within a regime where electronic energy losses are negligible relative to nuclear stopping power; thus, the primary damage state and defect clustering mechanisms are dominated by atomic collisions. Consequently, while the absolute Frenkel pair yield may be sensitive to the specifics of the high-energy potential, the qualitative trends in defect evolution and microstructure development—the central focus of this work—are expected to be reliably captured. Before irradiation simulations, the atomic configuration was energy-minimized using the conjugate gradient algorithm. The system was then equilibrated in the isothermal–isobaric ensemble at 300 K and ambient pressure for 100 ps under periodic boundary conditions to obtain a mechanically stable, stress-free initial state.

A sequence of 75 individual displacement cascades was initiated sequentially within the fixed simulation cell, following to the computational protocol outlined in Refs. [25,26]. For each cascade, a single PKA was randomly selected and assigned a kinetic energy of 5.0 keV, with its initial direction randomized. After the initial ballistic phase, the system’s evolution was tracked for about 85 ps using a variable time-step integration scheme, where the step size was dynamically adjusted between 10^−8^ and 10^−3^ ps. A boundary thermostat was applied to dissipate the excess heat generated by the PKA recoil, thereby maintaining the entire system at a constant temperature of 300 K throughout the annealing period. The chosen PKA energy of 5.0 keV corresponds to a characteristic value derived from the full PKA energy spectrum under MeV-neutron irradiation [15,19,26]. This energy scale is widely used in MD studies of primary radiation damage as it effectively captures the essential features of displacement cascade evolution while enabling the use of a computationally tractable simulation cell, thereby bridging simulation conditions with experimentally relevant damage states.

## 3. Results and Discussion

**Figure 2:**
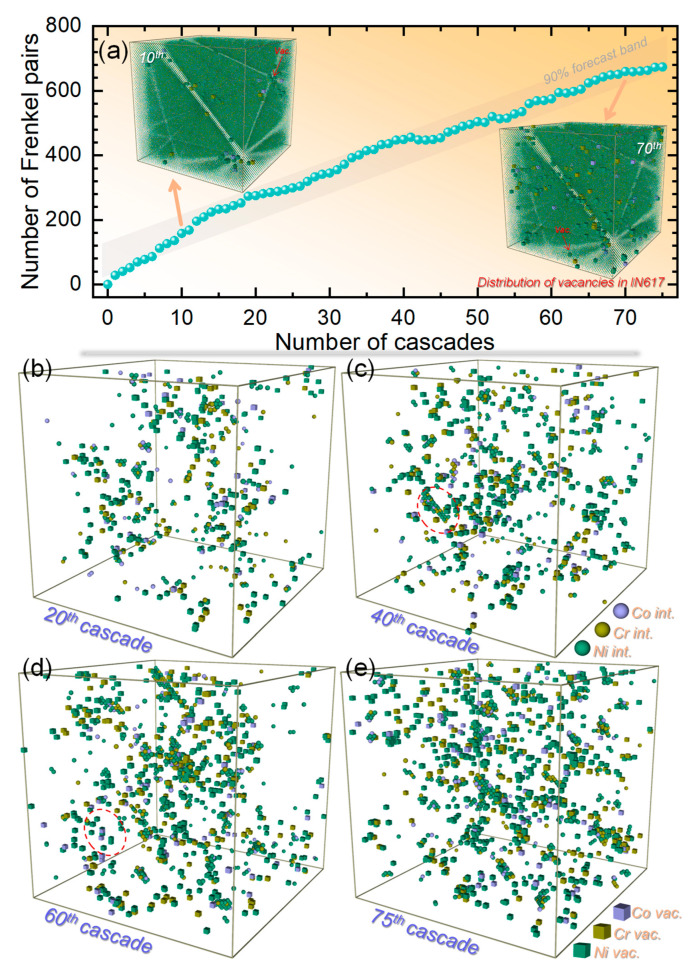
Evolution of point defects under sequential displacement cascades. (**a**) Number of surviving Frenkel pairs as a function of cascade count. (**b**–**e**) Atomic snapshots showing the distribution of surviving point defects (interstitials in ball, vacancies in cube) after 20 (**b**), 40 (**c**), 60 (**d**), and 75 (**e**) cascades.

Figure 2a shows the surviving Frenkel pair population in IN617 as a function of the cumulative number of displacement cascades, based on Wigner–Seitz (WS) analysis [16,26]. It should be noted that the WS method may systematically overestimate point defect numbers in close-packed metals owing to the delocalized nature of interstitial configurations. Nevertheless, it offers a consistent and computationally efficient means of tracking the relative accumulation and evolutionary trends of defects with increasing dose, which is the primary focus of this analysis. Accordingly, the reported defect populations should be interpreted as indicators of damage accumulation kinetics rather than as absolute experimental yields. The defect count rises steeply during the first 15 cascades, after which the net accumulation rate gradually decreases. This slowdown can be attributed to the increasing concentration of pre-existing defects, which enhances the probability of mutual annihilation between newly created and existing defects. Despite this, the overall trend exhibits a significant, nearly linear increase in Frenkel pair count with cascade progression, reflecting the continuous build-up of irradiation damage—a behavior frequently reported for both conventional and nanocrystalline nuclear materials [27,28,29,30] (e.g., Ni [28], Zr [30]) under sequential-cascade conditions. To spatially resolve defect evolution, atomic configurations after the 20, 40, 60, and 75 cascades are shown in Figure 2b–e. After 20 cascades, point defects associated with Ni, Cr, and Co are present in comparable quantities, even though Ni is the primary matrix element. Defects are distributed relatively uniformly, without marked clustering. Vacancies are predominantly isolated and rarely form clusters, whereas interstitials already exhibit noticeable aggregation. This divergence can be explained by the much lower migration barrier for interstitials compared to vacancies [19,31], which promotes rapid interstitial diffusion and clustering within the MD timeframe at 300 K, while vacancies remain comparatively immobile. By the 40th cascade, small vacancy clusters become more numerous, and interstitial clusters grow and coalesce into larger aggregates. Notably, many of these larger interstitial clusters adopt a preferential alignment along {111} or {112} crystallographic planes and tend to incorporate several Cr or Co atoms. This oriented arrangement indicates their evolution into precursors of dislocation loops. In fcc crystals, interstitial-type loops form most readily on the close-packed {111} planes, a configuration that minimizes elastic strain energy. Such crystallographic preference represents an intrinsic characteristic of the Ni–Cr–Co system under irradiation, governed by fundamental defect energetics rather than by prior thermomechanical processing. After 60 cascades, interstitial clustering progresses further, evolving from planar arrangements into three-dimensional aggregates that may act as precursors to interstitial-type dislocation loops. Clustering becomes more pronounced in the central region of the simulation cell at this stage. Some larger clusters observed after 40 cascades subsequently disappear (see dashed ellipses in Figure 2c,d), likely due to dissolution by subsequent cascade overlap. After 75 cascades, vacancies remain broadly dispersed and form only small clusters, even at higher doses, while interstitial clusters continue to grow. Throughout the irradiation sequence, an increasing fraction of Cr and Co atoms are displaced from lattice sites and become trapped within interstitial clusters—an effect that may pin these clusters and influence their long-term stability.

**Figure 3:**
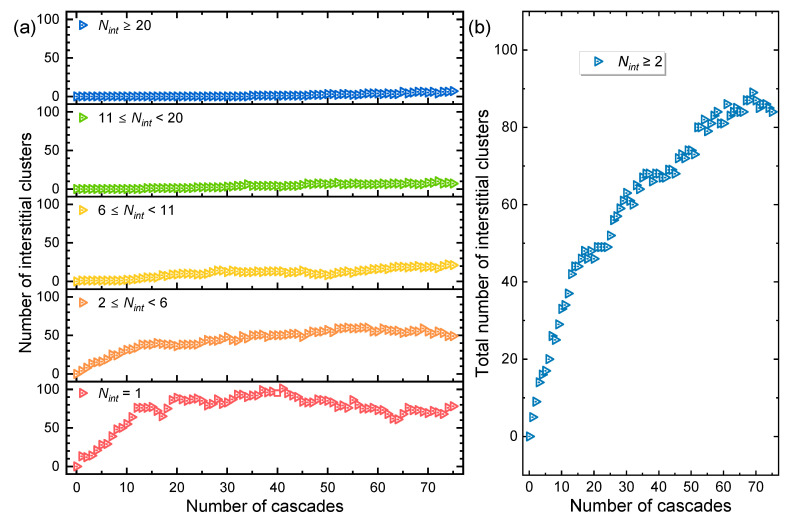
Evolution of interstitial clusters under sequential displacement cascades. (**a**) Number of interstitial clusters of different sizes (*N_int_*) as a function of cascade count. (**b**) Total number of interstitial clusters versus cumulative cascade count.

Sequential displacement cascades continuously introduce irradiation-induced point defects into the system. These defects subsequently accumulate and form clusters, with interstitial aggregation being particularly pronounced. To clarify the governing mechanisms, Figure 3a presents the size distribution of interstitial clusters at increasing dose. Clusters were defined using a cutoff radius based on the third-nearest-neighbor distance [32,33], with size expressed as *N_int_* (the number of interstitial atoms per cluster; e.g., *N_int_* = 3 for a tri-interstitial cluster). The evolution unfolds in three distinct stages. During the first 15 cascades, defects exist primarily as isolated interstitials and small clusters (2 ≤ *N_int_* ≤ 5), both populations increasing steadily. This trend reflects a dilute defect regime [34], where newly created Frenkel pairs are spatially separated within an otherwise undisturbed lattice. Between the 16th and 40th cascades, the number of isolated interstitials and small clusters (2 ≤ *N_int_* ≤ 5) continues to rise, but at a significantly slower rate, with the population of isolated interstitials saturating around the 40th cascade. Concurrently, medium (6 ≤ *N_int_* ≤ 10) and large clusters (*N_int_* ≥ 11) begin to emerge and grow. This shift indicates that the defect concentration has surpassed the dilute limit, enhancing interactions between point defects and promoting interstitial clustering. Furthermore, existing clusters start to capture newly formed interstitials or small clusters, signifying a transition in damage evolution from a nucleation-dominated regime to one governed by cluster growth and coalescence. From the 41st cascade onward, the number of isolated interstitials remains nearly constant, while the quantity of clusters of all sizes continues to rise steadily, including the appearance of clusters with *N_int_* ≥ 20. These observations imply that nearly all newly generated interstitials are incorporated into clusters. Since the total number of clusters increases only slowly, most interstitials are absorbed by pre-existing clusters, thereby coarsening the larger aggregates; only a minor fraction contributes to new cluster nucleation. Consistent with this, the total cluster count rises continuously with dose without reaching saturation (see Figure 3b), indicating that interstitial generation, migration, capture, and cluster interactions remain active throughout the simulated irradiation, and that established clusters do not completely inhibit the formation of new ones. The evolution of the interstitial cluster size distribution—characterized by an initial increase in small and medium clusters followed by the growth of large clusters at their expense—is governed by defect reaction kinetics and cluster thermodynamics. The high mobility of interstitials facilitates rapid nucleation of small, stable clusters during the thermal spike of each cascade. As the defect density increases, the system transitions from a nucleation-dominated regime to a coarsening regime. In this stage, larger clusters grow preferentially through two primary pathways: (1) the absorption of mobile single interstitials (and small clusters) generated in subsequent cascades, and (2) the dissolution and coalescence of smaller, less stable clusters via processes akin to Ostwald ripening. The significantly faster growth rate of large clusters compared to the decline of medium-sized clusters indicates that newly produced point defects are predominantly absorbed by existing large clusters. These larger clusters act as efficient sinks due to their lower defect formation energy per atom and greater stability.

**Figure 4:**
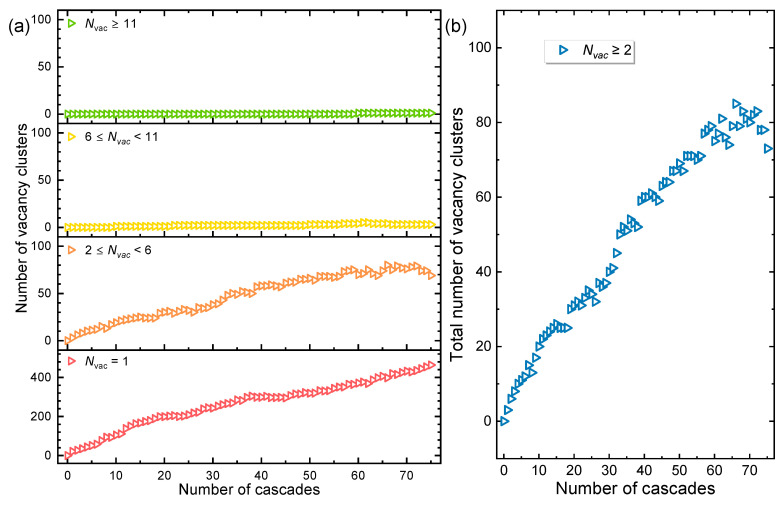
Evolution of vacancy clusters under sequential displacement cascades. (**a**) Number of vacancy clusters of different sizes (*N_vac_*) as a function of cascade count. (**b**) Total number of vacancy clusters versus cumulative cascade count.

In contrast to interstitials, vacancies display markedly different clustering behavior. The size distribution of vacancy clusters at increasing dose is presented in Figure 4a, where a cluster is defined using the second-nearest-neighbor distance as the cutoff [32,33], and its size is denoted by *N_vac_* (the number of vacancies it contains). Over the sequence of the overlapping cascades, the majority of vacancy defects—approximately two-thirds—persist as isolated single vacancies. The remainder predominantly forms small clusters, most containing between two and five vacancies. Both the isolated and small-cluster populations grow gradually with dose, whereas clusters with *N_int_* ≥ 6 are seldom observed. The increase in small vacancy clusters is both slower and less pronounced than that of isolated vacancies, underscoring the tendency of vacancies to remain dispersed. The scarcity of large vacancy clusters indicates that extensive vacancy aggregation is strongly inhibited under the simulated conditions. This is primarily due to the high migration energy of vacancies at 300 K, which severely restricts their mobility and coalescence within the MD timescale [35,36]. The limited number of larger clusters that do form originates mainly from regions of high vacancy density within individual cascade cores [37]; however, even with progressive cascade overlap, no significant acceleration in vacancy cluster growth is observed. Consequently, the variation in the total number of vacancy clusters as a function of cascade count (see Figure 4b) closely follows that of the small-cluster population (2 ≤ *N_int_* ≤ 5). Overall, these findings confirm that interstitials possess a far greater tendency to cluster than vacancies. While interstitials aggregate readily into large clusters, vacancies show little propensity to form into voids under the present conditions. This distinction aligns with established experimental evidence that irradiation hardening and embrittlement in metals below approximately 0.4T_m_ (where T_m_ is the melting temperature [38]) are driven primarily by the formation and interaction of interstitial-type dislocation loops [39,40]. It should be noted that these simulations were performed at 300 K to elucidate the ballistic formation and immediate evolution of primary damage. In Ni-based alloys, vacancy mobility is strongly suppressed at this temperature, thereby isolating the cascade-induced defect configurations. Under the elevated operational temperatures of an HP-MSR, significantly enhanced vacancy mobility would be expected to modify the long-term microstructural evolution, potentially promoting vacancy cluster growth and altering defect interaction kinetics. The primary damage state characterized here—particularly the pronounced tendency for interstitial clustering and solute (Cr/Co) trapping—provides the essential initial condition from which such temperature-dependent evolution would proceed.

**Figure 5:**
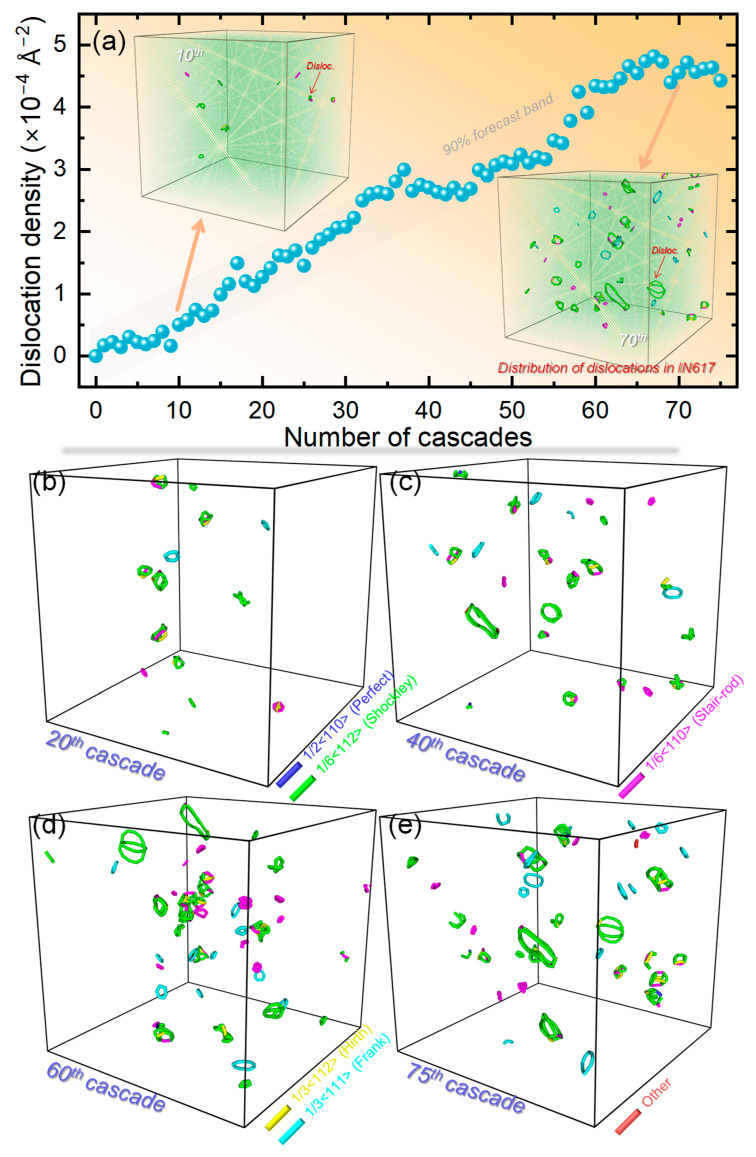
Evolution of dislocations under sequential displacement cascades. (**a**) Dislocation density as a function of cascade count. (**b**–**e**) Snapshots of the dislocation structures, extracted using the DXA technology, after 20 (**b**), 40 (**c**), 60 (**d**), and 75 (**e**) cascades. Different dislocation types are distinguished by color.

The mobility and coalescence of interstitials into substantial clusters promote favorable conditions for dislocation nucleation and subsequent evolution [41,42]. To investigate this process, Figure 5a plots the variation in dislocation density as a function of cascade number. Dislocation configurations and their temporal evolution were characterized using the Dislocation Extraction Algorithm (DXA) implemented in OVITO [21,26]. A near-linear rise in dislocation density is observed over the first 60 cascades, consistent with the expected progression from growing interstitial clusters to the collapse and formation of dislocation loops [41,43]. Despite minor fluctuations between the 35th and 60th cascades, the overall linear trend remains clear. After the 60th cascade, the density reaches a plateau with slight oscillations, indicating the onset of a dynamically saturated state where dislocation multiplication, cross-slip, climb, and annihilation processes achieve a steady-state equilibrium [44,45]. A more detailed breakdown of the dislocation network is presented in Figure 5b–e, which show the populations of different dislocation types after 20, 40, 60, and 75 cascades. Throughout the simulations, the network is dominated by Shockley partial dislocations with Burgers vectors of 1/6<112>, in agreement with typical stacking-fault and dissociation behavior in fcc crystals [46,47]. Smaller numbers of Stair-rod partials (1/6<110>), Hirth partials (1/3<110>), and Frank partials (1/3<111>) are also observed. The evolution of these populations reveals continuous reorganization of the dislocation structure. Up to the 60th cascade, all dislocation types increase in number with accumulating damage. Beyond this point, distinct trends emerge: the count of Shockley partials stabilize, Stair-rod partials decrease, while Hirth and Frank partials continue to increase gradually. This shift can be explained by reactive transformations, such as the conversion of Shockley and Stair-rod partials into Frank partials, suggesting that higher dislocation densities promote increased mutual interactions [48]. Consequently, certain dislocation configurations are either transmuted into other types or annihilated through reactions, driving continuous reconfiguration of the network. The pronounced tendency for interstitial-type dislocation formation observed here provides further support for existing experimental conclusions [38,39,40]. Additionally, the high density of interstitial clusters and dislocation loops, which act as strong obstacles to dislocation glide, offers a direct microstructural explanation for the irradiation hardening commonly reported in Ni-based alloys under similar conditions [49]. It should be noted that the present simulations utilize an ideal single-crystal model to elucidate the intrinsic defect evolution within the IN617 matrix. This serves as a necessary baseline, as the core mechanisms of interstitial clustering and solute trapping identified here will underpin damage evolution in more complex microstructures. In real polycrystalline IN617, microstructural features such as grain boundaries and pre-existing dislocations will act as potent defect sinks. It is well-established that grain boundaries can preferentially absorb interstitials due to their lower migration barrier, potentially reducing the intra-granular dislocation loop density observed here and affecting radiation-induced segregation profiles. Introducing these features in future simulation work will be critical for predicting mesoscale microstructural evolution and for guiding the design of grain-engineered materials with improved radiation tolerance. The insights gained from this bulk study provide an essential foundation for such multi-scale modeling efforts.

## 4. Conclusions

In summary, this study has employed classical MD simulations to investigate the irradiation-induced defect evolution in a ternary Ni–Cr–Co model representing IN617 alloy under sequential 5 keV displacement cascades at 300 K. The principal findings are concisely summarized as follows:

(1) The number of surviving Frenkel pairs increases in a near-linear manner with cumulative dose, with the net accumulation rate decreasing after the initial 15 cascades due to enhanced defect recombination at higher concentrations.
(2) A stark contrast in the behavior of point defects is observed. Interstitials exhibit high mobility, leading to the formation of large, stable three-dimensional clusters. In contrast, approximately two-thirds of vacancy defects remain as isolated point defects, with only minimal clustering due to their low mobility.
(3) A significant fraction of Cr and Co solute atoms are displaced from lattice sites and become trapped within the growing interstitial clusters, suggesting a potential solute pinning effect that may influence cluster stability.
(4) The coalescence of interstitial clusters facilitates the nucleation of dislocation loops, resulting in a linear increase in dislocation density that eventually saturates into a dynamic steady-state network after approximately 60 cascades. This network is predominantly composed of Shockley partial dislocations and undergoes continuous reorganization via reactions between partials.

These atomic-scale insights demonstrate that the microstructural response of IN617 under the simulated irradiation conditions is fundamentally governed by the aggressive clustering of interstitial defects, rather than vacancy void formation. This work provides the mechanistic understanding of the irradiation damage mechanisms in IN617, establishing a critical foundation for evaluating its microstructural stability and guiding its further development for advanced nuclear reactor applications.

## Data Availability

The original contributions presented in this study are included in the article. Further inquiries can be directed to the corresponding authors.
